# Rate of severe and fatal infections in a cohort of patients with interstitial lung disease associated with rheumatoid arthritis: a multicenter prospective study

**DOI:** 10.3389/fimmu.2024.1341321

**Published:** 2024-03-28

**Authors:** Natalia Mena-Vázquez, Rocío Redondo-Rodriguez, Marta Rojas-Gimenez, Carmen María Romero-Barco, Clara Fuego-Varela, Nair Perez-Gómez, Isabel Añón-Oñate, Patricia Castro Pérez, Aimara García-Studer, Ana Hidalgo-Conde, Rocío Arnedo Díez de los Ríos, Eva Cabrera-César, Maria Luisa Velloso-Feijoo, Sara Manrique-Arija, Jerusalem Calvo-Gutiérrez, Myriam Gandía-Martínez, Pilar Morales-Garrido, Francisco Javier Godoy-Navarrete, Coral Mouriño-Rodriguez, Francisco Espildora, María Carmen Aguilar-Hurtado, Antonio Fernández-Nebro

**Affiliations:** ^1^ Instituto de Investigación Biomédica de Málaga (IBIMA)-Plataforma Bionand, Málaga, Spain; ^2^ UGC de Reumatología, Hospital Regional Universitario de Málaga, Málaga, Spain; ^3^ Departamento de Medicina, Universidad de Málaga, Málaga, Spain; ^4^ Instituto Maimónides de Investigación Biomédica de Córdoba (IMIBIC), Córdoba, Spain; ^5^ UGC de Reumatología, Hospital Universitario Reina Sofía de Córdoba, Córdoba, Spain; ^6^ UGC de Reumatología, Hospital Clínico Universitario Virgen de la Victoria, Málaga, Spain; ^7^ UGC de Reumatología, Hospital Universitario de Jerez, Cádiz, Spain; ^8^ UGC de Reumatología, Complejo Hospitalario Universitario de Vigo, Vigo, Spain; ^9^ UGC de Reumatología, Hospital Universitario de Jaén, Jaén, Spain; ^10^ UGC de Reumatología, Hospital Universitario de Getafe, Madrid, Spain; ^11^ Servicio de Medicina Interna, Hospital Universitario Virgen de la Victoria, Málaga, Spain; ^12^ UGC Neumología, Hospital Universitario Virgen de la Victoria, Málaga, Spain; ^13^ UGC de Reumatología, Hospital Universitario Virgen de Valme, Sevilla, Spain; ^14^ UGC de Reumatología, Hospital Universitario Clínico San Cecilio, Granada, Spain; ^15^ UGC de Neumología, Hospital Regional Universitario de Málaga, Málaga, Spain; ^16^ UGC de Radiodiagnóstico, Hospital Regional Universitario de Málaga, Málaga, Spain

**Keywords:** rheumatoid arthritis, interstitial lung disease, infection, fatal infections microorganisms, inflammation

## Abstract

**Objective:**

To describe severe infection, foci of infection, microorganisms, associated factors, and impact on mortality in patients with rheumatoid arthritis–associated interstitial lung disease (RA-ILD).

**Patients and methods:**

The study was based on a multicenter prospective cohort of patients with RA-ILD followed up from 2015 to 2023. The main outcome measures were incident severe infection and fatal infection. We evaluated infectious foci, etiologic agents, vaccination status, variables associated with lung function, and clinical-therapeutic variables in RA. The incidence rate (IR) for infection and mortality was calculated per 100 person-years, and 3 multivariate models were constructed to explore factors associated with infection.

**Results:**

We followed up 148 patients with RA-ILD for a median 56.7 months (699.3 person-years). During this period, 142 patients (96%) had at least 1 infection. A total of 368 infectious episodes were recorded, with an IR of 52.6 per 100 person-years. Of the 48 patients who died, 65% did so from infection. Respiratory infections were the most common first infection (74%), infection overall (74%), and fatal infection (80%) and were caused mostly by SARS CoV-2*, Streptococcus pneumoniae, Pseudomonas aeruginosa*, and influenza A virus. The factors associated with an increased risk of infection and death in patients with RA-ILD were age, inflammatory activity, and therapy with corticosteroids and immunosuppressants.

**Conclusion:**

Patients with RA-ILD have a high risk of serious infection, especially respiratory infection. Infection develops early, is recurrent, and is frequently fatal. The presence of associated factors such as advanced age, joint inflammation, and treatment highlight the importance of integrated and preventive medical care.

## Introduction

Rheumatoid arthritis (RA) is a chronic systemic inflammatory autoimmune disease of unknown etiology that affects 0.5%-1% of the population. It is characterized mainly by symmetrical chronic synovitis affecting both small and large joints symmetrically. Without treatment, it leads to joint destruction and disability. Between 17.8% and 40.9% of patients with RA experience major extra-articular manifestations (1).

RA-associated interstitial lung disease (RA-ILD) is the most frequent pulmonary manifestation, with an incidence of between 4 and 4.5 cases per 1000 patient-years and a prevalence that varies widely between 1% and 58% (2). This broad variability is because a large percentage of RA patients have subclinical RA. In addition to its high frequency, RA-ILD has high morbidity and mortality (3), which seems to be increasing. Mortality in patients with RA-ILD has been reported to be almost 8 times greater than in other patients (4), and, in accordance with the study of Olson et al. (5), while all-cause mortality rates decreased between 1988 and 2004, the mortality of RA-ILD increased. In a systematic review aimed at determining the survival duration following RA-ILD diagnosis, it was found that pooled estimates for mortality were 9.0% (95% CI, 6.1-12.5) from more than 1 to ≤3 years and 49.1% (95% CI, 40.6-57.7) from more than 5 to ≤10 years. However, there was considerable heterogeneity among the study results (6).

There are many reasons why mortality is increased in patients with RA-ILD (3, 7–9). The associated factors include older age, male sex, more severely impaired lung function, and the usual interstitial pneumonia (UIP) radiologic pattern (7, 10). Similarly, more pronounced inflammatory activity and cardiovascular and respiratory factors have been associated with an unfavorable prognosis (11). Lastly, it is important to show that infection plays a key role in exacerbations and mortality in patients with RA-ILD. Kim et al. (2) found infection to be the cause of death in more than half of patients with RA-ILD, compared with only 16% in RA patients without ILD. Moreover, infections are the main cause of hospitalization in patients with RA-ILD treated with biologics or immunosuppressants, and superinfections are often associated with death resulting from disease progression (9, 12).

Patients with RA are more susceptible to infection, especially respiratory infection (13), owing to immune abnormalities caused by the disease, extra-articular manifestations such as ILD, and the treatment administered (14–16). This is also observed in idiopathic pulmonary fibrosis, in which the frequency of pulmonary infections increase owing to the underlying damage (16). While research on infections associated with RA-ILD is limited, it has been suggested that patients with both conditions may be at a 4.5-fold greater risk of infection (17–19) and that associated factors include disease duration and use of prednisone (20).

Studying infection in patients with RA-ILD is important when attempting to improve disease management. However, to date, there have been no in-depth studies on the main types of infection, risk factors, or the association with hospitalization and mortality. Therefore, given that knowledge of serious infection in patients with RA-ILD is lacking, we aimed to address 3 key objectives: 1) to describe the main pathogens causing infection; 2) to study the factors associated with the most common subtypes of infection; and 3) to analyze the association between hospitalization and mortality and infection.

## Patients and methods

### Design and data source

We performed a multicenter prospective observational study of a cohort of patients with RA-ILD from 11 university hospitals in Spain. Patients were recruited between March 2015 and March 2023. The study was approved by the Research Ethics Committee of Hospital Regional Universitario de Málaga (HRUM) (code: 1719-N-15). All participants provided their written informed consent before entering the study.

### Study population

The cases were included consecutively from a prospective cohort of patients with clinically significant RA-ILD. All the patients were adults and fulfilled the 2010 classification criteria for RA of the American College of Rheumatology/European League Against Rheumatism (21). ILD was confirmed based on pulmonary function testing (PFT) and high-resolution computed tomography (HRCT) or lung biopsy (22). We excluded patients with inflammatory or rheumatic disease other than RA (except secondary Sjögren syndrome), infection, primary pulmonary hypertension, congestive heart failure, and known exposure to fibrosing environmental agents. We also excluded pregnant women.

### Study protocol

The patients selected were seen by a rheumatologist in line with the data collection protocol at baseline (V0) and every 6-12 months, as well as at each admission for infection. Clinical and laboratory evaluations were performed for joint, lung, and infection-related variables. PFT and HRCT were performed at V0, and at 12, 24, and 60 months of follow-up or at any other time if the patient’s clinical situation so required in the opinion of the attending physician. The methodology used for performing and evaluating the HRCT and PFT in the study cohort have been described elsewhere (9, 23).

According to the protocol, coordinating meetings were held with the participants at baseline (V0), month 24 (V24), month 60 (V60), and at the end of follow-up (Vfinal). The data collection methodology was reviewed at each visit, and sufficient time was left to clarify doubts and resolve queries. In order to ensure the quality of data after collection, potential inconsistencies were analyzed at HRUM and queries that had to be corrected by the persons in charge at each center were noted.

### Infection-related outcomes

The main outcome was the incidence of serious and fetal infections. Fatal infection was defined as a death in which infection played a key role, either immediately or during the 30 days following the last admission for infection. Incident serious infection was defined as a condition caused by infectious agents that required antibiotic therapy and for which any of the following outcomes were recorded: death, a life-threatening condition, hospitalization (initial or prolonged), disability or permanent damage, congenital anomaly/birth defect, need for an intervention to prevent permanent impairment or damage, or other important medical events. The most common infectious sites in RA were recorded, especially those affecting the upper and lower respiratory tracts, the urinary tract, and skin and soft tissue (20, 24). The etiology of the infection was determined as follows (1): staining and isolation of the microorganism in blood culture, respiratory secretions, urine, stool, pleural or joint fluid, and tissue; and (2) molecular methods and antigen testing. The microorganism had to be compatible with the clinical findings in all cases (16). The presence of *Staphylococcus* species, *Streptococcus viridans* group, *Corynebacterium* species, *Propionibacterium* species, or *Bacillus* species in only 1 blood culture vial was considered contamination (25). The infection was considered to be of unknown origin when no pathogen was identified in culture or antigen tests (26). Patients’ vaccination status was also evaluated to specify whether they had received the full course of vaccination against pneumococci, influenza, COVID-19, and herpes zoster.

### Covariates associated with RA-ILD

The secondary outcome measures included baseline demographic data (sex, age, ethnic origin) and comorbidities (arterial hypertension, diabetes mellitus, dyslipidemia, obesity, and smoking history). The characteristics of RA included duration of RA, diagnostic delay, rheumatoid factor (reference value, 20 U/ml; high titer, > 60 U/ml), anti–citrullinated peptide antibodies (reference value, 10 U/ml, high values, ≥ 340 U/ml), radiological erosions, inflammatory activity according to the mean 28-joint Disease Activity Score with erythrocyte sedimentation rate (DAS28-ESR), C-reactive protein (CRP), and physical function according to the Health Assessment Questionnaire (HAQ). Mean DAS28-ESR was calculated as the mean of all DAS28-ESR values during follow-up. Similarly, we recorded the treatment used, including antibiotics, conventional synthetic disease-modifying antirheumatic drugs (csDMARDs), biologic DMARDs (bDMARDs), targeted synthetic DMARDs (tsDMARDs), immunosuppressants (azathioprine, mycophenolate mofetil, cyclophosphamide and tacrolimus), corticosteroids, and antifibrotic agents. In the case of corticosteroids, we collected the doses and mean dose during follow-up (27).

ILD was defined and classified according to the standard criteria of the American Thoracic Society/European Respiratory Society International Multidisciplinary Consensus Classification of the Idiopathic Interstitial Pneumonias (22). PFT included full spirometry expressed as percent predicted and adjusted for age, sex, and height. DLCO was evaluated using the single-breath method (DLCO-SB). Progression to ILD was categorized into the following outcomes (1): Improvement (i.e., improvement in forced vital capacity (FVC) ≥ 10% or diffusing capacity of the lungs for carbon monoxide (DLCO) ≥ 15% and absence of radiological progression) (2); Nonprogression (stabilization or improvement in FVC ≤ 10% or DLCO < 15% and absence of radiological progression) (3); Progression (worsening of FVC > 10% or DLCO > 15% and radiological progression); or (4) Death (9).

### Statistical analysis

We performed a descriptive analysis of the main characteristics of patients with RA-ILD and of the type and number of infections, the initial infection, and associated types of infection in patients who died. Qualitative variables were expressed as absolute number and percentage; quantitative variables were expressed as mean (standard deviation [SD]) or median (p25-p75), depending on the normality of the distribution according to the Kolmogorov-Smirnov test. Data were missing in 10/148 patients (6.7%) for DAS28-ESR and in 42/148 patients (28.4%) for the HAQ. The patients with missing data were not excluded in order to maintain a representative cohort; however, they were managed based on imputation of data using regression analysis for the descriptive analysis; this approach involves specifying predictor variables related to the missing values and running a regression analysis to predict and fill in the missing values (28). Quantitative variables were compared using the *t* test for independent variables or the Wilcoxon–Mann-Whitney test; qualitative variables were compared using the Pearson χ^2^ test. A bivariate analysis was performed with the paired *t* test or Wilcoxon test, as applicable, between V0 and Vfinal for lung and joint function. The risk of infection or death was estimated using incidence rates and their respective 95% confidence intervals (95%CI), using the Poisson method (29). The incidence rate for infection and death was calculated by dividing the number of events detected by the “time at risk” of the cohort. The “time at risk” was calculated in person-years by summing the times each patient had remained in observation between V0 and death and the date of the end of the observation period (Vfinal, March 2023 – censored case), since no losses to follow-up were recorded. Factors associated with infection up to the time of the first severe infection were identified using uni- and multivariate Cox regression analysis. Survival was measured from V0 to Vfinal or death. Furthermore, we ran uni- and multivariate multiple linear regression models to identify factors associated with the number of infections. The statistical analyses were performed using IBM SPSS Statistics for Macintosh, Version 28.0 (IBM Corp., Armonk, NY, USA).

## Results

### Baseline clinical characteristics

A total of 148 patients with RA-ILD were prospectively followed up between March 2015 and March 2023. Mean (SD) follow-up was 56.7 (22.4) months, that is, a total cumulative exposure of 699.3 patient-years. The main characteristics at baseline (V0) are shown in **Table 1**. The average age was 70 years, and 57% were women. A total of 51.4% of the patients had never smoked, while the remaining 48.6% had a history of smoking. The most comorbid condition was arterial hypertension (48%) followed by dyslipidemia (37%) and osteoporosis (37%). Most patients had seropositive disease (98%) with only 3/148 patients (2%) seronegative disease, and approximately 60% had erosions.

**Table 1:** Baseline characteristics of patients with RA-ILD.

Variable	N = 148
Epidemiologic characteristics	
Female sex, n (%)	84 (56.8)
Caucasian race, n (%)	143 (96.6)
Age in years, mean (SD)	69.0 (9.6)
Comorbid conditions	
Dyslipidemia, n (%)	56 (37.8)
Arterial hypertension, n (%)	72 (48.6)
Smoking	
Nonsmoker, n (%)	76 (51.4)
Smoker, n (%)	27 (18.2)
Exsmoker, n (%)	45 (30.4)
Diabetes mellitus, n (%)	25 (16.9)
History of cardiovascular disease, n (%)	24 (16.2)
Chronic obstructive pulmonary disease, n (%)	15 (10.1)
Congestive heart failure, n (%)	13 (11.8)
Peptic ulcer, n (%)	17 (11.5)
Liver disease, n (%)	11 (7.4)
Moderate-severe kidney disease, n (%)	20 (6.8)
Cancer, n (%)	11 (7.4)
Osteoporosis, n (%)	56 (37.8)
Clinical and laboratory characteristics	
Time since diagnosis of RA, months, median (p25-p75)	140.9 (68.9-221.1)
Diagnostic delay, months, median (p25-p75)	7.3 (4.2-12.8)
Time since diagnosis of ILD, months, median (p25-p75)	32.5 (15.9-53.5)
Positive RF (>10 IU/ml), n (%)	143 (96.6)
Positive ACPA (>20 IU/ml), n (%)	129 (87.2)
Erosive disease, n (%)	88 (59.5)
Treatment	
Conventional synthetic DMARD	122 (82.4)
Methotrexate, n (%)	60 (40.5)
Leflunomide, n (%)	37 (25.0)
Sulfasalazine, n (%)	9 (6.1)
Hydroxychloroquine, n (%)	29 (19.6)
Biologic DMARD	81 (54.7)
Infliximab, n (%)	1 (0.7)
Etanercept, n (%)	7 (4.7)
Adalimumab, n (%)	3 (2.0)
Golimumab, n (%)	3 (2.0)
Certolizumab, n (%)	3 (2.0)
Tocilizumab, n (%)	6 (4.1)
Abatacept, n (%)	39 (26.4)
Rituximab, n (%)	19 (12.8)
Immunosuppressants	28 (18.9)
Mycophenolate, n (%)	23 (15.5)
Azathioprine, n (%)	5 (3.4)
Antifibrotics, nintedanib n (%)	2 (1.4)
Corticosteroids, n (%)	106 (71.6)
Dose of corticosteroids, median (p25-p75)	5.0 (0.0-7.5)

RA, rheumatoid arthritis; ILD, interstitial lung disease; SD, standard deviation; RF, rheumatoid factor; ACPA, anti–citrullinated peptide antibodies; DMARD, disease-modifying antirheumatic drug.

At baseline (V0), the average duration of ILD among patients was 2.7 years. The most common radiologic pattern was UIP (90/148 patients [60.4%]), followed by NSIP (45/148 [30.2%]), fibrotic NSIP (11/148 [7.4%]), and other types of ILD (3/148 patients [2%]).

In terms of treatment, all the patients were receiving a DMARD at V0. Most were receiving csDMARDs and slightly more than half were receiving bDMARDs. At the initiation of the study, 67 patients (45%) were receiving csDMARD monotherapy, 58 (39%) were receiving a combination of csDMARDs and bDMARDs, 23 (16%) bDMARD monotherapy, 19 (13%) a combination of a csDMARD and an immunosuppressant, and a further 15 (10%) a combination of a bDMARD and an immunosuppressant. Only 1 patient (1.4%) was receiving a csDMARD plus nintedanib. The DMARDs prescribed at V0 are shown in **Table 1**. Furthermore, we can see that more than half the patients were taking corticosteroids, with a median (p25-p75) dose of 5.0 (0.0-7.5) mg/d.

### Clinical characteristics during follow-up

At the last evaluation, 100 patients were still in follow-up, whereas 48 patients had died. There were no losses to follow-up. **Table 2** shows the initial data (V0) and final data (Vfinal) for lung and joint function. A significant deterioration was observed in forced vital capacity (FVC), forced expiratory volume in the first second (FEV_1_), and DLCO-SB at Vfinal. Of the first 148 patients, 66 (44.6%) experienced radiologic progression according to their HRCT scan, 78 (52.7%) had stabilized, and only 4 (2.7%) improved. Evaluation of the joints revealed no significant differences in inflammatory activity (DAS28-ESR and CRP) or in physical function evaluated using the HAQ.

**Table 2:** Progress of joint and lung variables in 148 patients with RA-ILD.

Variable	Baseline	End of follow-up	p Value
Duration of follow-up, months, mean (SD)	–	56.7 (22.4)	–
Pulmonary function			
Oxygen saturation (%), mean (SD)	96.2 (2.0)	95.4 (3.1)	0.005
Pulmonary function testing			
FVC (%), mean (SD)	75.5 (16.2)	66.7 (18.2)	<0.001
FEV_1_ (%), mean (SD)	80.7 (16.0)	71.6 (17.4)	<0.001
DLCO-SB (%), mean (SD)	59.1 (14.8)	54.4 (15.9)	<0.001
HRCT pattern			
UIP, n (%)	89 (60.1)	107 (72.3)	
NSIP, n (%)	44 (29.7)	26 (17.6)	
Fibrotic NSIP, n (%)	11 (7.4)	11 (7.4)	
Other types, n (%)	4 (2.7)	4 (2.7)	
Radiological findings			
Progression, n (%)	–	66 (44.6)	
Stabilization, n (%)	–	78 (52.7)	
Improvement, n (%)	–	4 (2.7)	
Progression of lung disease*			
Improvement, n (%)	–	5 (3.4)	
Stabilization, n (%)	–	59 (39.9)	
Worsening, n (%)	–	36 (24.3)	
Death, n (%)	–	48 (32.4)	
Activity of RA			
DAS28, median (p25-p75)	2.8 (2.3-4.0)	3.0 (2.4-3.5)	0.271
CRP (mg/L), median (p25-p75)	5.9 (2.9-13.0)	6.1 (3.1-17.0)	0.714
HAQ, median (p25-p75)	1.0 (0.2-1.5)	1.0 (0.5-2.0)	0.206

*Progression of lung disease: considering HRCT and PFT (FVC and DLCO).

RA, rheumatoid arthritis; ILD, interstitial lung disease; SD, standard deviation; FVC, forced vital capacity; FEV_1_, forced expiratory volume in the first second; DLCO, diffusing capacity of the lungs for carbon monoxide; HRCT, high-resolution computed tomography; UIP, usual interstitial pneumonia; NSIP, nonspecific interstitial pneumonia; DAS28, 28-joint Disease Activity Score; CRP, C-reactive protein; HAQ, Health Assessment Questionnaire; PFT, pulmonary function testing.

As can be seen in **Supplementary Table 1**, at the end of follow-up (Vfinal), 45 patients had switched therapy owing to arthritis and/or lung disease. These changes were characterized mainly by an increase in the frequency of bDMARDs in monotherapy (10.8% *vs* 18%; p=0.019), together with reduced use of methotrexate (40.5% *vs* 30.2%; p=0.004), leflunomide (25% *vs* 20.1%; p=0.057), and etanercept (4.7% *vs* 1.3%; p=0.025) in favor of increased prescription of abatacept (26.4% *vs* 32%; p=0.060), rituximab (12.8% *vs* 15.4%; p=0.103), and nintedanib (1.4% *vs* 5.4%; p=0.014).

### Incidence of infection and mortality

**Figure 1:**
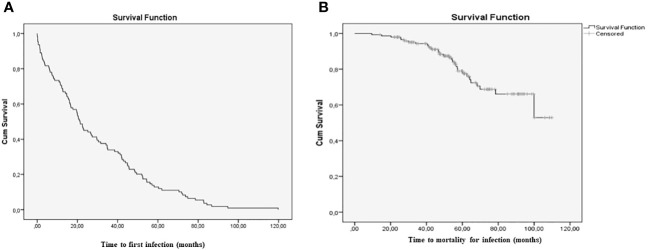
Survival curves; **(A)** Time to the first infection; **(B)** Time to death from infection.* The image include RA-ILD patients who developed infection.

During the follow-up period, 142/148 patients (96%) had at least 1 infection, with a median (p25-p75) time to first infection of 21.2 (8.0-45.2) months (**Figure 1A**). As can be seen in the survival graph, the risk of infection remained constant throughout the follow-up period. We recorded a total of 368 infectious episodes in the 148 patients, that is, a median (p25-p75) of 2 infections (1.0-3.0) per patient and an incidence rate (95% CI) of 52.6 (47.2-58.0) per 100 person-years. A direct association was observed between age and the probability of infection during follow-up. Notably, the incidence rate of infections per 100 person-years increased notably among patients aged 50 and older. The rates were 49.0 (95% CI: 38.0-60.0) in the 50-59 years age group, 44.0 (95% CI: 34.0-52.0) in the 60-69 years age group, 55.0 (95% CI: 45.0-63.0) in the 70-79 years age group and peaked at 62.0 (95% CI: 44.0-80.0) in the 80-89 years age group (**Supplementary Table 2**).

**Figure 2:**
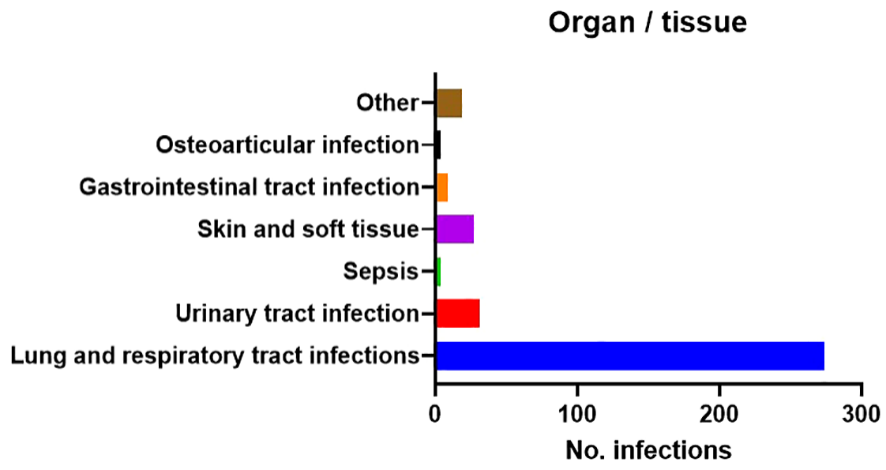
Number and type of infections.

**Table 3** and **Figure 2** show details of the number and type of infections, as well as the vaccination status of the 148 patients with RA-ILD. Respiratory infections were the most prevalent (274/368 [85.8%]); other sites were involved in 94/368 infections (25.5%), the most frequent being the urinary tract, followed by the skin and soft tissue.

**Table 3:** Frequency and types of infection and vaccination status in 148 patients with RA-ILD.

Variable	N=148
Infection, n (%)	142 (95.9)
Number of infections, median (p25-p75)	2.0 (1.0-3.0)
IR of infection (95% CI) × 100 p-y	52.6 (47.2-58.0)
Types of infection	
Respiratory infection, n (%)	127 (85.8)
Upper airway, n (%)	83 (56.1)
Lower airway, n (%)	62 (41.9)
COVID-19, n (%)	32 (21.6)
Other, n (%)	61 (41.2)
Urinary tract, n (%)	26 (17.6)
Skin and soft tissue, n (%)	24 (16.2)
Gastrointestinal, n (%)	9 (6.1)
Septic arthritis, n (%)	1 (0.7)
Osteomyelitis, n (%)	3 (2.0)
Herpes zoster, n (%)	4 (2.7)
Dental, n (%)	6 (4.0)
Sepsis, n (%)	4 (2.7)
Eye, n (%)	4 (2.7)
Otitis, n (%)	5 (3.4)
Genital infection, n (%)	2 (1.4)
IR respiratory infection (95% CI) × 100 p-y	40.9 (36.1-45.8)
IR other infections (95% CI) × 100 p-y	13.4 (10.7-16.1)
Deaths from infection, n (%)	31 (20.9)
Respiratory infection, n (%)	25 (16.8)
Upper airway, n (%)	0 (0.0)
Lower airway, n (%)	17 (11.4)
COVID-19, n (%)	8 (5.0)
Other, n (%)	6 (4.0)
Urinary tract infection, n (%)	1 (0.7)
Skin and soft tissue, n (%)	1 (0.7)
Gastrointestinal, n (%)	1 (0.7)
Sepsis, n (%)	3 (2.0)
IR death from infection (95% CI) × 100 p-y	4.4 (2.8-5.9)
Pneumococcal vaccine, n (%)	139 (93.9)
Influenza vaccine, n (%)	143 (96.6)
COVID-19 vaccine, n (%)	126 (85.1)
Herpes zoster vaccine, n (%)	5 (3.4)

RA, rheumatoid arthritis; ILD, interstitial lung disease; IR, incidence rate; CI, confidence interval; p-y, person-years.

Most patients had been appropriately vaccinated against influenza, pneumococci, and COVID-19; only 5 patients had been vaccinated against herpes zoster virus. Patients who were not vaccinated experienced a higher incidence of infections compared to those who were vaccinated (50% *vs* 24.6%; p=0.015), as well as a higher number of deaths (50% *vs* 14.3%; p=0.034). Out of 32 patients with COVID-19 infection, 10 received corticosteroids, 5 were treated with azithromycin, 4 with levofloxacin, 3 with ceftriaxone, 2 received a combination of azithromycin and levofloxacin, 1 was prescribed amoxicillin, 1 received septrim/paxlovid, 1 was treated with azithromycin/paxlovid, and 1 received ceftriaxone/paxlovid.

A total of 48 patients died during follow-up. Of these, 31 (65%) died directly from infection as the main cause: 80% from respiratory infection and 20% from other types of infection (**Table 3**). The remaining 17/48 patients (35%) died from noninfectious causes: 14/48 mainly due to progression of lung disease, 1 from brain hemorrhage, and 2 from cancer (lung adenocarcinoma and primary brain tumor).

**Table 4** and **Supplementary Table 3** provide a detailed description of the causative microorganisms, number of infections, incidence rate classed by totals, first infection, infections with an identified pathogen, and deaths from infection. Respiratory infection was the most common first infection (74%), followed by urinary tract infection (9.9%) and skin and soft tissue infection (9.1%). There were no cases of ENT infection or sepsis as the first infection. Of the 31 patients who died from infection, all had had a respiratory infection during follow-up, mainly affecting the lower respiratory tract. All patients who died from infection did so during their last admission for infection, except 1 patient, who died during the 30 days following discharge (readmission). Compared with the remaining patients (ie, those who were still alive or had died of other causes), those who died from infection had a higher median (p25-p75) for the number of previous infections (3.0 [2.0-5.0] *vs* 2.0 [1.0-3.0]; p<0.001) and a higher incidence of infection (incidence rate [95%CI] 84.39 [68.34-100.46] per 100 person-years *vs*. 45.74 [40.20-51.28] per 100 person-years; incidence rate ratio [95%CI] 1.84 [1.48-2.30]) (**Figure 1B**).

**Table 4:** Number of infections and incidence of infection stratified by totals, first infection, infections with an identified microorganism, and deaths from infection.

Organ/tissue	All infections (N=368)		Infections with identified microorganism (N=134)	First infection (N=142)		Deaths from infection (N=31)	
	N (%)	IR (95% CI) x 100 p-y	N (%)	N (%)	IR (95% CI) x 100 p-y	N (%)	IR (95% CI) x 100 p-y
Lung and respiratory tract	274 (74.5)	39.18 (34.54-43.82)	100 (79.1)	106 (74.6)	15.16 (1227-18.04)	25 (80.6)	3.57 (22.17-4.98)
Urinary tract	31 (8.4)	4.43 (2.87-5.99)	17 (12.7)	14 (9.9)	2.00 (0.95-3.05)	1 (0.7)	0.14 (0.00-0.42)
Skin and soft tissue	27 (7.3)	3.86 (2.40-5.32)	8 (6.0)	13 (9.1)	1.86 (048-2.87)	1 (0.7)	0.14 (0.00-0.42)
Gastrointestinal tract	9 (2.4)	1.29 (0.45-2.13)	3 (2.2)	3 (2.1)	0.43 (0.00-0.91)	1 (0.7)	0.14 (0.00-0.42)
Septic arthritis	1 (0.3)	0.14 (0.00-0.42)	1 (0.7)	1 (0.7)	0.14 (0.00-0.42)	0 (0.0)	—
Osteomyelitis	3 (0.8)	0.43 (0.00-0.91)	1 (0.7)	1 (0.7)	0.14 (0.00-0.42)	0 (0.0)	—
Sepsis	4 (1.1)	0.57 (0.00- 1.13)	2 (1,5)	0 (0.0)	—	3 (2.0)	0.43 (0.00-0.91)
Eye and orbit	6 (1.6)	0.86 (0.17-1.54)	0	1 (0.7)	0.14 (0.00-0.42)	0 (0.0)	—
Oral and dental	6 (1.6)	0.86 (0.17-1.54)	1 (0.7)	2 (1.4)	0.19 (0.00-0.68)	0 (0.0)	—
ENT	5 (1.3)	0.71 (0.10-1.34)	0	0 (0.0)	—	0 (0.0)	—
Genital tract	2 (0.5)	0.19 (0.00-0.68)	1 (0.7)	1 (0.7)	0.14 (0.00-0.42)	0 (0.0)	—

RA, rheumatoid arthritis; ILD, interstitial lung disease; IR, incidence rate; ENT, ear, nose, and throat.

With respect to the causative microorganisms (**Table 4**, **Supplementary Table 3**), the pathogen was not identified in a significant number of cases (234/368 [63.5%]); this result was similar for all 3 scenarios: total number of infections, number with a first infection, and deaths from infection. Given the total number of infections in which the causative microorganism was identified (n=134), most were caused by SARS CoV-2 (33.5%), *Streptococcus pneumoniae* (11.9%), *Escherichia coli* (11.9%), and *Pseudomonas aeruginosa* (11.1%). These microorganisms were also the most common in the first infection, together with *Klebsiella pneumoniae*. Furthermore, in the 31 patients who died, the most fatal microorganisms were SARS CoV-2 (25.8%), *P. aeruginosa* (12.9), and pneumococci (9.6%), followed by *K. pneumoniae* (3.2%), *Haemophilus influenzae* (3.2%), influenza A virus (3.2%), and respiratory syncytial virus (3.2%). It is worth noting that of the 8 deaths caused by SARS CoV-2, 6 (75%) were during 2020 and 2021. While no patients died from an opportunistic infection, the causative agent was not identified in 38% of fatal infections. Finally, patients who died from infection received a much higher median dose of corticosteroids than patients who survived (5.0 [5.0-15.0] *vs*. 0.0 [0.0-5.0], p < 0.001).

### Factors associated with infection and mortality

In the multivariate Cox regression models adjusted for duration of follow-up (**Table 5**), we assessed various factors in patients with RA-ILD to understand their impact on the risk of first infection and death by infection. Age, inflammatory activity, and daily corticosteroid dosage showed as significant predictors for both outcomes. For first infections, age (hazard ratio [HR]=1.030, p = 0.011), DAS28 (HR=1.211, P=0.037), and corticosteroid dosage (HR=1.036, p = 0.012) were significant. Regarding death by infection, age (HR =1.108, p < 0.001), DAS28 (HR=1.497, P=0.037), DLCO-SB (HR=0.975, p = 0.044), and corticosteroid dosage (HR=1.065, p = 0.001) showed significance.

**Table 5:** Multivariate Cox regression analysis.

Predictor	Univariate		Multivariate		
	HR	95% CI	HR	95% CI	p Value
First infection*					
Female sex	0.897	0.638,1.263			
Age	1.117	1.004, 1.037	1.030	1.007, 1.054	0.011
Smoking	1.029	0.823, 1.288			
ACPA	1.299	0.780, 2.164			
Erosions	1.132	0.805, 1.591			
FVC	0.996	0.980, 1.007			
DLCO-SB	0.994	0.981, 1.005			
UIP pattern	0.921	0.726, 1.170			
Mean DAS28	1.158	1.001, 1.340	1.211	1.012, 1.450	0.037
CRP (mg/L)	1.008	1.000-1.071			
csDMARDs	1.055	0.687, 1.619			
bDMARDs	1.539	0.886, 2.673			
Immunosuppressants	1.064	0.693, 1.633			
Corticosteroids, mg/d	1.030	1.004, 1.056	1.036	1.008, 1.064	0.012
Death by infection*					
Female sex	1.526	0.714, 3.263			
Age	1.114	1.060, 1.171	1.108	1.052, 1.167	<0.001
Smoking	1.075	0.674, 1.715			
ACPA	1.573	0.478, 5.180			
Erosions	1.436	0.674, 3,056			
FVC	0.968	0.946, 0.990			
DLCO-SB	0.962	0.940, 0.985	0.975	0.951, 0.999	0.044
UIP pattern	1.093	0.694, 1.722			
Mean DAS28	1.393	1.074, 1.805	1.497	1.024, 2.190	0.037
CRP (mg/L)	1.008	0.991, 1.025			
csDMARDs	0.783	0.336, 1.828			
bDMARDs	0.915	0.452, 1.854			
Immunosuppressants	0.473	0.144, 1.557			
Corticosteroids, mg/d	1.092	1.054, 1.131	1.065	1.025, 1.107	0.001

*Cox regression analysis adjusted for time since diagnosis of ILD.

Variables included in the equation: sex, age, smoking, ACPA, erosions, FVC, DLCO, UIP pattern, mean DAS28, CRP, csDMARDs, bDMARDS, immunosuppressants, corticosteroid dose.

RA, rheumatoid arthritis; ILD, interstitial lung disease; ACPA, anti–citrullinated peptide antibody; FVC, forced vital capacity; DLCO, diffusing capacity of the lungs for carbon monoxide; DAS28, 28-joint Disease Activity Score; CRP, C reactive protein; UIP pattern, UIP pattern vs rest; csDMARD, conventional synthetic disease-modifying antirheumatic drug; Bdmard, biologic disease-modifying antirheumatic drug.

On the other hand, **Table 6** shows the results of a multiple linear regression model of predictors for the number of infections in RA-ILD patients. Notably, time with ILD (B = 0.012, p = 0.043), mean DAS28 (B = 0.268, p = 0.009), treatment with immunosuppressants (B = 0.670, p = 0.042), and the dose of corticosteroids (B = 0.095, p < 0.001) showed significant associations with the number of infections.

**Table 6:** Multivariate linear regression analysis.

Predictor	Univariate		Multivariate		
	B	95% CI	B	95% CI	p Value
Number of infections					
Female sex	0.347	-0.202, 0.897			
Age	0.016	-0.013, 0.045			
Time with ILD	0.014	0.002, 0.026	0.012	0.001, 0.023	0.043
Smoking	0.309	-0.056, 0.673			
ACPA	0.936	0.088, 1.784			
Erosions	-0.305	-0.886, 0.276			
FVC	-0.003	-0.021, 0.016			
DLCO	-0.012	-0.033, 0.009			
UIP pattern	0.211	-0.131, 0.553			
Mean DAS28	0.245	0.005, 0.485	0.268	0.040, 0.496	0.009
csDMARDs	0.101	-0.559, 0.760			
bDMARDs	0.368	-0.246, 0.983			
Immunosuppressants	0.723	0.049, 1.397	0.670	0.024, 1.315	0.042
Dose of corticosteroids	0.094	0.055, 0.133	0.095	0.057, 0.134	<0.001

Variables included in the equation: sex, age, smoking, ACPA, erosions, FVC, DLCO, UIP pattern, mean DAS28, csDMARDs, bDMARDs, immunosuppressants, dose of corticosteroids, time since diagnosis of ILD.

RA, rheumatoid arthritis; ILD, interstitial lung disease; ACPA, anti–citrullinated peptide antibody; FVC, forced vital capacity; DLCO, diffusing capacity of the lungs for carbon monoxide; DAS28, 28-joint Disease Activity Score; UIP pattern, UIP pattern vs rest; csDMARD, conventional synthetic disease-modifying antirheumatic drug; bDMARD, biologic disease-modifying antirheumatic drug.

## Discussion

In our study, we performed an exhaustive and relevant analysis of the burden of serious infections in a large multicenter cohort of 148 patients with RA-ILD over a long period. The scarcity of data in this area constitutes a major knowledge gap. Our results show that almost all patients with RA-ILD (96%) had at least 1 serious infection, thus demonstrating a considerable risk of experiencing events of this type. The incidence of serious infection was 52.6 (47.2-58.0) per 100 person-years. Despite the limited availability of similar studies, the frequency we report is higher than that reported elsewhere for serious infections in patients with RA-ILD (20, 24, 30), probably, in part, because of our exhaustive, prospective patient follow-up. For example, Zamora-Legoff et al. (20) found the incidence of serious infection in patients with RA-ILD to be 7.4 per 100 person-years. However, the study was retrospective, only included patients hospitalized with infection, and did not record fatal infections. Similarly, Sugano et al. (30) reported serious infections in 26% of patients from an urban cohort with RA-ILD evaluated retrospectively over 5 years based on twice-yearly surveys. The study specifically analyzed patients who had not achieved remission according to DAS28. Moreover, the main objective of the study was not to analyze patients with RA-ILD.

One particularly relevant finding of our analysis was that the median time to first infection was 21 months, thus highlighting the early vulnerability of this patient population to infection. Furthermore, each patient had an average of 2 infections during the study period, underscoring the constant susceptibility to recurrent infections in this population.

As for the distribution of the infections reported, respiratory infection was the most frequent type as first infection and as infection overall, followed by urinary tract infection and skin and soft tissue infection. This finding coincides with previously reported data (18, 20, 30–32), where the predominance of respiratory infection in this population could be due to the functional and structural damage affecting the lung. However, a novel contribution of our study was the identification and comparison of the microorganisms causing the infection. On the one hand, we showed that the causative agent could not be identified in a significant percentage of infections (63%), indicating that diagnosis of the infection continues to be challenging. On the other, the most frequent pathogens identified were respiratory viruses, mainly SARS CoV-2, and *S. pneumoniae*, followed by *E. coli*, *P. aeruginosa*, and influenza A virus. While no similar studies have been performed to date, other studies on respiratory infections in immunodepressed patients report that infection is caused mainly by conventional bacteria (40%), followed by viruses, fungi, and, albeit to a lesser extent, *P. jiroveci* and *M. tuberculosis*. Furthermore, the pathogen remains unidentified in a variable percentage of patients (33–35).

Another notable aspect of our study is the special attention paid to infection-related mortality in patients with RA-ILD. The association between infection and mortality is of paramount importance. Our results indicate that approximately 65% of deaths during follow-up were directly related to infection. These results are consistent with those reported in the study by Kim et al. (2), who found that, despite the reduced number of deaths, infection was the cause of death in 66% of patients with RA-ILD.

Among the different types of infection, serious respiratory infection was also associated with higher mortality in our cohort (80%). In this sense, we highlight that the most fatal pathogens were SARS CoV-2 (25.8%), *P. aeruginosa* (12.9%), and pneumococci (9.6%). While are unable to make direct comparisons with other studies, these pathogens have been reported to have a more serious impact on patients with RA-ILD (9, 31). Therefore, it is worth noting that the observation period in our study included the most critical points during the COVID-19 pandemic. This would explain why one third of all infections and one quarter of fatal infections were caused by SARS CoV-2 and why 6 of the 8 deaths caused by this virus were during 2020 and 2021.

Our study also revealed the key factors associated with risk of infection and fatal infection in patients with RA-ILD to be age, activity of arthritis, and treatment with corticosteroids. The relationship between age and risk of infection has been reported elsewhere in the general population and in patients with RA (2, 36, 37), potentially reflecting the influence of a weakened immune system in older patients and greater susceptibility to infections resulting from treatment (37). In relation to this, we observed that the incidence rate of infections per 100 person-years increased significantly among patients aged 50-59, with rates of 49.0 (95% CI: 38.0-60.0), reaching a peak of 62.0 (95% CI: 44.0-80.0) in the 80-89 years age group. Furthermore, the relationship between activity of arthritis and risk of infection could be linked to the immune dysfunction associated with the underlying autoimmune disease, increased acute phase reactant values, and treatment itself, especially, prednisone (15, 20). These findings highlight the crucial role of age, inflammation, and corticosteroid dosage in influencing infection risks and mortality in RA-ILD patients.

In addition, in an alternative model examining the number of infections in RA-ILD patients, inflammatory activity and higher corticosteroid doses demonstrated a significant association with increased infection rates. Additionally, time with ILD duration and the use of immunosuppressants were also identified as contributing factors to a higher incidence of infections. Similarly, the duration and degree of lung involvement suggest that the severity and progression of ILD could have a significant impact on the risk of infection (9, 38). These results emphasize the relevance of disease duration, disease activity, and treatment in influencing infection frequency among RA-ILD patients.

The present study has both strengths and weaknesses, and both must be taken into account when interpreting the results. In terms of the reliability and validity of the data, we highlight the prospective design and the fact that the sample included all cases of clinically significant RA-ILD. This selection strategy ensured more accurate results by guaranteeing adequate representation of the population of interest. Moreover, appropriate management of confounders lends rigor to the methodology and strengthens the internal validity of the study. As described in material and methods, data were missing in 10/148 patients (6.7%) for DAS28-ESR and in 42/148 patients (28.4%) for the HAQ; however, they were managed based on imputation of data using regression analysis for the descriptive analysis (28). Moreover, while data loss is a frequent occurrence in prospective cohort studies, the extent of this loss has been minimal and had no impact on the outcomes. The prospective observational design of the study prevented us from controlling allocation of treatment. Since treatments were based on routine clinical practices rather than being randomly allocated, definitively determining whether the outcomes of infections are directly linked to the treatment or involve other factors becomes difficult. However, a study design based on daily clinical practice provides more practical data that can be applied in the real world. It is also important to note that our definition of serious infection was that used in clinical trials and most observational studies. Nevertheless, we consider that our definition was clear and simple, making it possible to describe the outcomes used in clinical practice with accurate data that could be compared with data from other studies. Moreover, we discussed the impact of the COVID-19 pandemic on our findings. This could be seen as both a limitation and a strength since recent research on COVID-19 suggests that previous waves of SARS-CoV-2 infections indicate the virus is likely to become endemic, with sporadic resurgences (39). This affords us a more realistic and up-to-date understanding of COVID-19 infections in patients with RA-ILD. Finally, it is relevant to note that our study did not include a control group, thus limiting our ability to see the causal relationship between ILD and the clinical outcomes analyzed. However, the main strength of our study is that it is the first to report the frequency of a number of infections and the pathogens that affect patients with RA-ILD.

In conclusion, we provide new data on the burden of severe infection in a cohort of patients with RA-ILD. Our results demonstrate a high occurrence of serious infections among these patients, occurring early, recurring frequently, and proving fatal in 65% of cases. Respiratory infection was the main infection in all the clinical scenarios examined, namely, first infection, total infections, and mortality, where the main causative agents were SARS CoV-2, *S. pneumoniae*, *P. aeruginosa*, and influenza A virus. Factors such as age, the inflammatory activity of arthritis, and treatment with corticosteroids and immunosuppressants were associated with a greater risk of infection and mortality in patients with RA-ILD. Our results highlight the need for an integrated approach to manage and prevent these complications. We hope that our findings serve as the basis for future studies and improve the care provided to and quality of life of this vulnerable population.

## Data availability statement

The original contributions presented in the study are included in the article/**Supplementary Material**. Further inquiries can be directed to the corresponding author.

## Ethics statement

The study was approved by the Research Ethics Committee of Hospital Regional Universitario de Málaga (HRUM) (code: 1719-N-15). The studies were conducted in accordance with the local legislation and institutional requirements. The participants provided their written informed consent to participate in this study.

## Author contributions

NM-V: Conceptualization, Methodology, Writing – original draft. RR-R: Investigation, Writing – original draft. MR-G: Investigation, Writing – review & editing. CR-B: Investigation, Writing – review & editing. CF-V: Investigation, Writing – review & editing. NP-G: Investigation, Writing – review & editing. IA-O: Investigation, Writing – review & editing. PP: Investigation, Writing – review & editing. AS: Investigation, Writing – review & editing. AH: Investigation, Writing – review & editing. RR: Investigation, Writing – review & editing. EC: Investigation, Writing – review & editing. MV-F: Investigation, Writing – review & editing. SM-A: Investigation, Writing – review & editing. JC-G: Investigation, Writing – review & editing. MG-M: Investigation, Writing – review & editing. PM-G: Investigation, Writing – review & editing. FG-N: Investigation, Writing – review & editing. CM-R: Investigation, Writing – review & editing. FE: Investigation, Writing – review & editing. MA-H: Investigation, Writing – review & editing. AF-N: Investigation, Supervision, Writing – original draft.
