# Supplementary material for: Rate of severe and fatal infections in a cohort of patients with interstitial lung disease associated with rheumatoid arthritis: a multicenter prospective study
Source: Front Immunol. 2024 Mar 28;15:1341321. doi: 10.3389/fimmu.2024.1341321 (PMC11007097; doi:10.3389/fimmu.2024.1341321)
Supplement: Supplementary file 1 [file Table_1.docx]

Supplementary table 1: Changes in treatment during the follow-up of 148 patients with RA-ILD.

| Variable | Baseline | End of follow-up | p Value |
| --- | --- | --- | --- |
| Treatment |  |  |  |
| Conventional synthetic DMARD | 122 (82.4) | 103 (69.6) | 0.001 |
| Methotrexate, n (%) | 60 (40.5) | 45 (30.2) | 0.004 |
| Leflunomide, n (%) | 37 (25.0) | 30 (20.1) | 0.057 |
| Sulfasalazine, n (%) | 9 (6.1) | 8 (5.4) | 0.960 |
| Hydroxychloroquine, n (%) | 29 (19.5) | 29 (19.5) | 1.000 |
| Biologic DMARD | 81 (54.7) | 84 (56.8) | 0.607 |
| Infliximab, n (%) | 1 (0.7) | 0 (0.0) | 0.319 |
| Etanercept, n (%) | 7 (4.7) | 2 (1.3) | 0.025 |
| Adalimumab, n (%) | 3 (2.0) | 1 (0.7) | 0.158 |
| Golimumab, n (%) | 3 (2.0) | 3 (2.0) | 1.000 |
| Certolizumab, n (%) | 3 (2.0) | 1 (0.7) | 0.158 |
| Tocilizumab, n (%) | 6 (4.1) | 6 (4.0) | 1.000 |
| Abatacept, n (%) | 39 (26.4) | 48 (32.0) | 0.060 |
| Rituximab, n (%) | 19 (12.8) | 23 (15.4) | 0.103 |
| Targeted synthetic DMARD, n (%) | 0 (0.0) | 2 (1.3) | 0.149 |
| Baricitinib, n (%) | 0 (0.0) | 1 (0.7) | 0.319 |
| Filgotinib, n (%) | 0 (0.0) | 1 (0.7) | 0.319 |
| Immunosuppressant | 28 (18.9) | 27 (18.1) | 0.797 |
| Mycophenolate, n (%) | 23 (15.5) | 22 (14.8) | 0.809 |
| Azathioprine, n (%) | 5 (3.4) | 5 (3.4) | 1.000 |
| Antifibrotic agents, nintedanib n (%) | 2 (1.4) | 8 (5.4) | 0.014 |
| Corticosteroids, n (%) | 106 (71.6) | 111 (75.0) | 0.166 |
| Doses of corticosteroid, median (p25-p75) | 5.0 (0.0-7.5) | 5.0 (2.5-7.5) | 0.882 |
| Combinations |  |  |  |
| csDMARD in monotherapy, n (%) | 54 (36.5) | 48 (32.4) | 0.286 |
| bDMARD in monotherapy, n (%) | 16 (10.8) | 26 (18.0) | 0.019 |
| IS in monotherapy, n (%) | 2 (1.4) | 3 (2.0) | 0.500 |
| tsDMARD in monotherapy, n (%) | 0 (0.0) | 1 (0.7) | - |
| csDMARD + bDMARD | 49 (33.1) | 42 (29.0) | 0.093 |
| csDMARD + IS | 10 (6.8) | 7 (4.7) | 0.508 |
| bDMARD + IS | 7 (4.7) | 10 (6.8) | 0.508 |
| csDMARD + bDMARD + IS | 8 (5.4) | 2 (1.4) | 0.031 |
| tsDMARD + IS | 0 | 1 (0.6) | - |
| csDMARD + nintedanib | 1 (0.6) | 2 (1.4) | 0.501 |
| bDMARD + nintedanib | 1 (0.6) | 2 (1.4) | 0.501 |
| csDMARD + IS + nintedanib | 0 (0.0) | 2 (1.4) | - |
| bDMARD + IS+ nintedanib | 0 (0.0) | 1 (0.7) | - |
| IS+ nintedanib | 0 (0.0) | 1 (0.7) | - |

*Abbreviations. RA: rheumatoid arthritis; ILD: interstitial lung disease; DMARD: disease-modifying antirheumatic drug; csDMARD: conventional synthetic disease modifying antirheumatic drug; bDMARD: biological DMARD; tsDMARD: targeted synthetic DMARD; IS: immunosuppressant.*

Supplementary table 2: Incidence rate of infection by age group

| Age group | Number of patients | Incidence rate of infections (95% CI) per 100 person-years |
| --- | --- | --- |
| 20-29 years | 0 | - |
| 30-39 years | 0 | - |
| 40-49 years | 2 | 11.0 (2.0-16.0) |
| 50-59 years | 25 | 49.0 (38.0-60.0) |
| 60-69 years | 41 | 44.0 (34.0-52.0) |
| 70-79 years | 62 | 55.0 (45.0-63.0) |
| 80-89 years | 18 | 62.0 (44.0-80.0) |
| >90 years | 0 | - |

Supplementary table 3: Microorganisms causing infection (total), first infections, and infection-related mortality.

| Microorganism | Site affected | Total no. of infections | No. with a first infection | Deaths |
| --- | --- | --- | --- | --- |
| *Pseudomonas aeruginosa* | Joint | 1 | 1 | 0 |
|  | Sepsis | 1 | 0 | 1 |
|  | Respiratory tract | 13 | 0 | 3 |
| *Haemophilus influenzae* | Respiratory tract | 4 | 2 | 1 |
| *Escherichia coli* | Urinary tract | 16 | 5 | 0 |
| *Streptococcus pneumoniae* | Respiratory tract | 16 | 8 | 3 |
| *Herpes zoster* | Skin | 4 | 2 | 0 |
| *Klebsiella pneumoniae* | Respiratory tract | 3 | 3 | 1 |
|  | Urinary tract | 1 | 1 | 0 |
| SARS CoV-2 | Respiratory tract | 45 | 14 | 8 |
| *Candida albicans* | Oral cavity | 1 | 1 | 0 |
| *Staphylococcus aureus* | Skin and soft tissue | 3 | 3 | 0 |
| *Streptococcus* | Sepsis | 1 | 0 | 0 |
|  | Respiratory tract | 1 | 0 | 0 |
|  | Skin | 1 | 1 | 0 |
| Enterobacteria | Respiratory tract | 1 | 1 | 0 |
|  | Osteomyelitis | 1 | 1 | 0 |
| Influenza A virus | Respiratory tract | 7 | 1 | 1 |
| *Pneumocystis jiroveci* | Respiratory tract | 3 | 1 | 0 |
| Hepatitis B virus | Gastrointestinal | 1 | 1 | 0 |
| *Helicobacter pylori* | Gastrointestinal | 1 | 0 | 0 |
| *Aspergillus* | Respiratory tract | 3 | 0 | 0 |
| Human papillomavirus | Genital | 1 | 0 | 0 |
| Respiratory syncytial virus | Respiratory tract | 2 | 0 | 1 |
| *Campylobacter jejuni* | Respiratory tract | 1 | 0 | 0 |
| *Clostridium difficile* | Gastrointestinal | 1 | 0 | 0 |
| Tuberculosis | Respiratory tract | 1 | 0 | 0 |
